# Kerosene Dangers

**Published:** 1871-09

**Authors:** 


### KEROSENE DANGERS.

Many lives are lost every year, perhaps a life a day, in the employment of this very useful article, from carelessness, ignorance, or inattention. A woman was burned to death recently by attempting to blow the light out, downwards, through the top of the glass chimney attached to the lamp; the flame was forced down in contact with the oil. The flame should be extinguished by stooping down, and blowing upwards.

Explosions frequently take place by allowing the wick to burn until the supply of oil is nearly exhausted; if the oil was not allowed to burn but half way down, these explosions would never take place.

Attempting to pour in oil while the lamp is burning, is a frequent cause of fatal results.

As the fumes of the oil become concentrated, when the vessel containing it is kept well stopped, they often take fire at the instant of uncorking, from a burning light being near. The safest, best, and cleanliest way, is to trim lamps out of doors, and in the daytime.

